# QuickStats

**Published:** 2014-02-07

**Authors:** FROM THE NATIONAL CENTER FOR HEALTH STATISTICS

**Figure:**
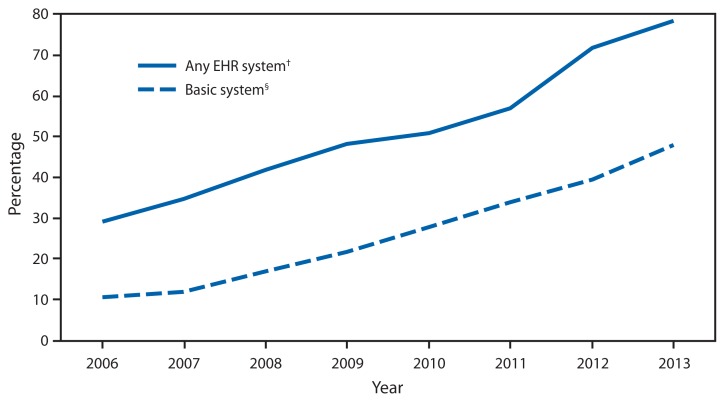
Percentage of Office-Based Physicians with Electronic Health Record (EHR) Systems — National Ambulatory Medical Care Survey,* United States, 2006–2013 * A sample survey of office-based physicians. ^†^ A medical or health record system that is either all or partially electronic. ^§^ A system with the following functionalities: patient history and demographics, patient problem lists, physician clinical notes, comprehensive list of patient medications and allergies, computerized orders for prescriptions, and the ability to view laboratory and imaging results electronically.

During 2006–2013, the percentage of physicians using any EHR system increased 168%, from 29.2% in 2006 to 78.4% in 2013. Nearly half of physicians (48.1%) were using the more comprehensive “basic system” by 2013, up from 10.5% in 2006.

**Source:** Hsiao CJ, Hing E. Use and characteristics of electronic health record systems among office-based physician practices: United States, 2001–2013. NCHS data brief no. 143. Hyattsville, MD: US Department of Health and Human Services, CDC; 2014. Available at http://www.cdc.gov/nchs/data/databriefs/db143.pdf.

**Reported by:** Esther Hing, MPH, ehing@cdc.gov, 301-458-4271; Chun-Ju Hsiao, PhD.

